# Investigating the associations between adiposity, life course overweight trajectories, and telomere length

**DOI:** 10.18632/aging.101036

**Published:** 2016-09-18

**Authors:** Wahyu Wulaningsih, Johnathan Watkins, Tetsuya Matsuguchi, Rebecca Hardy

**Affiliations:** ^1^ Medical Research Council Unit for Lifelong Health and Ageing at UCL, University College London, London WC1B 5JU, UK; ^2^ Division of Haematology/Oncology, Faculty of Medicine, Universitas Gadjah Mada, Yogyakarta 55292, Indonesia; ^3^ PILAR Research and Education, Cambridge CB1 2JD, UK; ^4^ Department of Biochemistry and Biophysics, University of California, San Francisco, CA 94158, USA; ^5^ Driver Group, L.L.C., San Francisco, CA 94158, USA

**Keywords:** obesity, overweight, life course, telomere, ageing, BMI

## Abstract

Obesity may accelerate ageing through chronic inflammation. To further examine this association, we assessed current adiposity, adiposity at early adulthood and life course overweight trajectories in relation to leukocyte telomere length (LTL). We included a total of 7,008 nationally representative U.S. residents and collected information on objectively measured body mass index (BMI), waist circumference and percent body fat. BMI at age 25 and overweight trajectories were assessed using self-reported history. Leukocyte telomere length (LTL) relative to a standard DNA reference (T/S ratio) was quantified by polymerase chain reaction (PCR). Linear regression models were used to examine the difference in LTL across adiposity measures at examination, BMI at age 25, and overweight trajectories. A 0.2% decrease in telomere length (95% CI:−0.3 to −0.07%) was observed for every kg/m2 increase in BMI, whereas a unit increase in waist circumference (cm) and percent body fat contributed to a 0.09% and 0.01% decrease in LTL, respectively. Higher BMI and being obese at age 25 contributed to lower LTL at older ages. Associations between weight loss through life course and LTL were observed, which further marked the importance of life course adiposity dynamics as a determinant of ageing.

## INTRODUCTION

Obesity continues to be an expanding health problem in both low-, middle- and high-income countries [1–3]. Estimates suggest that the global prevalence of obesity will reach 18% in men and surpass 21% in women by 2025 if current trends continue [4]. Individuals with excess adiposity, as indicated by measures of body mass index (BMI) [5, 6] and waist circumference [7], are at higher risk of chronic disease including diabetes and cardiovascular disorders. Obesity-induced endoplasmic reticulum (ER) stress, which leads to various metabolic and immune dysregulation, may link excess adiposity to the development of these age-related diseases [8, 9], ER stress has also been linked with cellular senescence [10], further implying a role for obesity in ageing.

Several observational studies have investigated the association between obesity and ageing though assessment of telomere length. The telomere refers to terminal DNA-protein complexes at the chromosome, which primarily functions to maintain chromosomal stability [11]. Telomere shortening is regarded a marker of biological ageing and has been linked with higher risk of cardiovascular disease and death [12, 13]. However, the association between obesity and telomere length remains elusive. In a recent meta-analysis including a total of twelve studies and 8,010 participants, a 16% lower standardised mean difference (SMD) of telomere length was observed cross-sectionally in obese individuals compared to those with normal weight (Summary SMD: 0.84, 95% CI: 0.22–1.46), but this finding was not statistically significant [14]. There was also high heterogeneity among studies which was not explained by laboratory methods used to assess telomere length. Moreover, most included studies used BMI to define obesity, with central obesity only measured in two Asian studies [15, 16] and body fat in one British study excluding individuals with BMI 30 kg/m2 or above [17]. There is indication that adiposity in earlier age, or its life course trajectories, may impact physiological functions in later life as shown with blood pressure [18]. However, little is known regarding any impact of adiposity at earlier age or changes of adiposity over time on telomere length at later life, especially in the general population.

To gain further insight into the role of obesity in ageing, we characterised the association between adiposity measures including BMI, waist circumference and body fat, and telomere length in the 1999-2002 U.S. National Health and Nutrition Examination Survey (NHANES). Furthermore, we additionally assessed telomere length using self-reported information on BMI at age 25 among those 30 years and older. An understanding of the lifetime relationships between obesity and telomere length may help guide public health efforts to reduce levels of obesity and its adverse health outcomes.

## RESULTS

Weighted characteristics of study participants and corresponding mean relative telomere length are shown in Table 1. Telomere length (LTL) decreased with older age, shortest among non-Hispanic whites and those who did not finish high school. The mean BMI measured at examination in men and women were 27.8 ± 0.14 and 28.1 ± 0.20 kg/m2, respectively, and the mean self-reported BMI was 27.7 ± 0.15 and 27.3 ± 0.18 kg/m2 for men and women, respectively. Over 65% of the participants had measured BMI of 25 kg/m2 or greater, and around 46% of them were obese. Nearly 48% of participants had excess waist circumference, and 59% of participants had excess body fat.

**Table 1:** Characteristics of study participants and mean telomere length (LTL)

	N	Weighted %	Mean LTL (95% CI)
**Age, years**			
20-29	1107	17.84	1.21 (1.17-1.24)
30-39	1182	21.33	1.11 (1.08-1.15)
40-49	1288	22.15	1.07 (1.04-1.11)
50-59	975	16.52	0.99 (0.96-1.03)
60-69	1169	11.00	0.96 (0.92-0.99)
70-85	1287	11.16	0.87 (0.84-0.90)
**Sex**			
Male	3586	49.70	1.05 (1.05-1.08)
Female	3422	50.30	1.06 (1.03-1.09)
**Race/ethnicity**			
Non-Hispanic white	3581	74.02	1.04 (1.01-1.08)
Non-Hispanic black	1219	9.19	1.11 (1.07-1.15)
Mexican American	1683	6.89	1.06 (1.02-1.09)
Other	525	9.90	1.10 (1.04-1.17)
**Education**			
Less than high school	2360	21.01	1.02 (0.99-1.05)
High school	1639	26.24	1.05 (1.01-1.09)
Higher education	3009	52.75	1.08 (1.05-1.10)
**Smoking status**			
Current smokers	3525	49.74	1.07 (1.04-1.11)
Former smokers	1901	25.46	1.01 (0.98-1.04)
Never smokers	1582	24.80	1.07 (1.04-1.11)
**Alcohol consumption**			
Never	950	11.86	1.06 (1.02-1.11)
Up to once a week	4677	65.25	1.05 (1.02-1.08)
2-3 times per week	696	12.07	1.09 (1.06-1.13)
4 times per week or more	685	10.82	1.04 (1.01-1.07)
**Vigorously active**			
No	5867	80.25	1.04 (1.01-1.07)
Yes	1141	19.75	1.12 (1.09-1.14)
**Cancer diagnosis**			
No	6418	92.17	1.06 (1.03-1.09)
Yes	590	7.83	0.97 (0.93-1.00)
**Diabetes**			
No	5978	89.47	1.07 (1.04-1.10)
Yes	1030	10.53	0.98 (0.95-1.01)
**Hypertension**			
No	3965	63.50	1.09 (1.06-1.12)
Yes	3043	36.50	1.00 (0.97-1.03)
**Hypertriglyceridemia**			
No	5782	83.41	1.07 (1.04-1.10)
Yes	1226	16.59	1.00 (0.96-1.04)
**Low HDL**			
No	4458	63.55	1.06 (1.03-1.09)
Yes	2550	36.45	1.05 (1.02-1.08)

### Associations between current adiposity and telomere length

When assessing objectively measured adiposity and LTL at current age, we found shorter LTL with higher measured BMI, waist circumference, and percent body fat. In the fully adjusted model, a 0.2% decrease in telomere length (95% CI: −0.3 to −0.06%) was observed for every increase in BMI, and a unit increase in waist circumference and percent body fat contributed to a 0.08% (95% CI: −0.1 to−0.03%) and 0.2% (-0.3 to−0.03%) decrease in LTL, respectively. Trends with adiposity categories were similar. Being obese (BMI 30 kg/m2 or greater) was associated with a 3% (95% CI: −5 to−0.9%) decrease in LTL compared to those with BMI 18.5-25 kg/m2. With the dichotomous overweight status (</≥25 kg/m2), we found that being overweight was associated with a 3% decrease in LTL (95% CI:−4 to−0.3%). Estimates were similar for excess adiposity by waist circumference and percent body fat (Table 2). Results did not markedly alter after we adjusted for presence of other metabolic disorders and cancer diagnosis.

**Table 2:** Associations between current adiposity measures and log-transformed leukocyte telomere length (LTL)

	**N**	Weighted %	Mean LTL (SE)	Log LTL difference^1^		Log LTL difference^2^		Log LTL difference^2^,^3^	
				β	95% CI	β	95% CI	β	95% CI
**BMI, kg/m^2^**									
Continuous				−0.002	−0.004, −0.0007	−0.002	−0.003, −0.0006	−0.002	−0.003, −0.0007
Clinical cut-off									
<18.5	104	17.54	1.07 (0.03)	−0.04	−0.08, 0.005	−0.03	−0.07, 0.01	−0.03	−0.07, 0.01
18.5-25	2137	33.07	1.09 (0.02)	Reference		Reference		Reference	
25-30	2556	35.09	1.04 (0.02)	−0.02	−0.04, 0.002	−0.02	−0.04, 0.002	−0.02	−0.04, 0.004
≥30	2211	30.08	1.03 (0.02)	−0.04	−0.06, −0.01	−0.03	−0.05, −0.009	−0.03	−0.05, −0.01
**Waist circumference^4^**									
Continuous				−0.001	−0.002, −0.0004	−0.0008	−0.001, −0.0003	−0.0009	−0.001, −0.0003
Clinical cut-off									
Normal	3437	52.11	1.09 (0.02)	Reference		Reference		Reference	
High	3571	47.89	1.02 (0.02)	−0.03	−0.05, −0.009	−0.02	−0.04, −0.006	−0.02	−0.04, −0.006
**Percent body fat^5^**									
Continuous				−0.002	−0.003, −0.0004	−0.002	−0.003, −0.0003	−0.001	−0.003, −0.00005
Clinical cut-off									
Normal	1122	40.46	1.16 (0.02)	Reference		Reference		Reference	
High	1746	59.54	1.11 (0.02)	−0.03	−0.05, −0.01	−0.03	−0.05, −0.009	−0.02	−0.04, −0.005

1 Adjusted for age at LTL measurement (continuous), sex, and race/ethnicity

2 Adjusted for age at LTL measurement (continuous), sex, race/ethnicity, education, smoking status, alcohol intake, vigorous physical activity

3 Additional adjustment for diabetes, hypertension, hypertriglyceridemia, low HDL, and self-reported cancer diagnosis

4 Normal: ≤102 cm (men) and ≤88 cm (women); high: >102 cm (men) and >88 cm (women)

5 Measured in 2,868 individuals aged 20-49 years. Normal: ≤25% (men) and ≤35% (women); high: >25% (men) and >35%

### Quantile regression analysis

**Figure 1:**
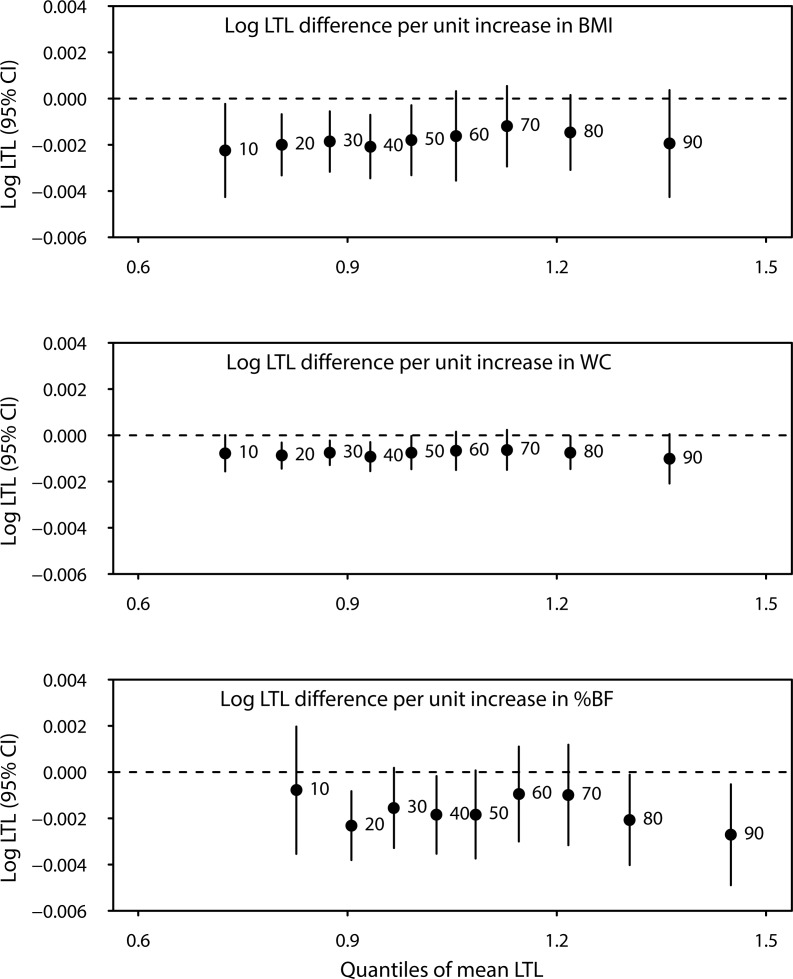
Difference in mean log-transformed LTL (T/S ratio) across quantiles of LTL for every unit increase in body mass index (BMI), waist circumference (WC), and percent body fat (%BF) All models were adjusted for age at LTL measurement (continuous), sex, race/ethnicity, education, smoking status, alcohol intake, and vigorous physical activity.

We further performed quantile regression analyses to characterise the association between adiposity measures and LTL. This analysis provided information on changes in extreme quantiles and outliers of LTL for every unit increase in adiposity indices. Changes in LTL with increasing adiposity varied across LTL quantiles as shown in Figure 1. However, no statistically significant difference across quantiles overall were found with Wald test (P=0.15, 0.45 and 0.62 for BMI, waist circumference and percent body fat, respectively). Associations with the median (50^th^ quantile) of LTL were similar to those observed using the linear regression approach, with 0.2%, 0.08% and 0.01% decreases in LTL with every unit increase in BMI, waist circumference and percent body fat, respectively

All models were adjusted for age at LTL measurement (continuous), sex, race/ethnicity, education, smoking status, alcohol intake, and vigorous physical activity.

### Self-reported adiposity in relation to current telomere length

We further assessed adiposity history using self-reported BMI and LTL. Table 3 showed associations between self-reported BMI at current age and at age 25 with LTL at current age. Associations between self-reported current BMI and LTL were similar to those observed with objectively measured BMI. In age-stratified analyses, a 0.5% decrease in LTL was observed among those aged 30-40 for every unit increase in BMI at age 25 years. A similar association was seen between being obese at 25 years and LTL at age 30-40. Among those aged 50-60, a 7% decrease in LTL was seen with higher annual change in BMI since aged 25 years. A test for interaction suggested a statistically significant interaction between BMI at age 25 and age group (P_interaction_=0.004). Associations were similar when we used the estimation of measured BMI at age 25 from regressing current measured over self-reported BMI (Table 3).

**Table 3:** Associations between self-reported BMI and log-transformed leukocyte telomere length (LTL) All models were adjusted for age at LTL measurement (continuous), sex, race/ethnicity, education, smoking status, alcohol intake, and vigorous physical activity.

Age at examination	N (%)	Log LTL difference		Log LTL difference^1^	
		β	95% CI	β	95% CI
**Current BMI, kg/m^2^**					
**All ages**					
Continuous		-0.002	-0.003, -0.0003	-0.003	-0.004, -0.0003
Clinical cut-off					
<18.5	90 (1.51)	-0.06	-0.12, 0.002	-0.005	-0.10, 0.09
18.5-25	2283 (35.96)	Reference		Reference	
25-30	2540 (35.86)	-0.03	-0.04, -0.01	-0.02	-0.04, -0.0003
≥30		-0.04	-0.06, -0.02	-0.03	-0.06, -0.01
**BMI at age 25, kg/m^2^**					
**All ages^2^**					
Continuous		-0.002	-0.004, -0.0004	-0.002	-0.004, -0.0004
Clinical cut-off					
<18.5	104 (1.75)	0.007	-0.02, 0.03	0.02	-0.02, 0.05
18.5-25	2137 (33.07)	Reference		Reference	
25-30	2556 (35.09)	-0.008	-0.03, 0.01	-0.004	-0.03, 0.02
≥30	2211 (30.08)	-0.03	-0.06, 0.001	-0.02	-0.05, 0.008
Annual change^3^		-0.02	-0.03, -0.004	-0.02	-0.03, -0.005
**Age 30-39**					
Continuous		-0.005	-0.008, -0.003	-0.005	-0.008, -0.003
Clinical cut-off					
<18.5	60 (5.23)	0.04	-0.03, 0.06	0.04	-0.03, 0.11
18.5-25	676 (60.28)	Reference		Reference	
25-30	299 (23.95)	-0.02	-0.08, -0.003	-0.02	-0.06, 0.02
≥30	122 (10.54)	-0.05	-0.11, -0.04	-0.05	-0.09, -0.02
Annual change^3^		-0.003	-0.03, 0.02	-0.005	-0.03, 0.02
**Age 40-49**					
Continuous		-0.002	-0.007, 0.002	-0.002	-0.007, 0.002
Clinical cut-off					
<18.5	75 (5.96)	-0.02	-0.08, 0.04	-0.03	-0.13, 0.05
18.5-25	780 (63.63)	Reference		Reference	
25-30	298 (23.54)	-0.02	-0.06, 0.03	-0.01	-0.06, 0.04
≥30	99 (6.87)	-0.03	-0.08, 0.04	-0.01	-0.08, 0.05
Annual change^3^		-0.08	-0.18, 0.007	-0.08	-0.19, 0.004
**Age 50-59**					
Continuous		0.002	-0.001, 0.005	0.002	-0.001, 0.005
Clinical cut-off					
<18.5	79 (8.52)	-0.001	-0.06, 0.06	0.03	-0.05, 0.13
18.5-25	628 (66.89)	Reference		Reference	
25-30	193 (20.13)	0.04	-0.001, 0.08	0.02	-0.02, 0.06
≥30	51 (4.46)	0.02	-0.04, 0.09	0.02	-0.03, 0.08
Annual change^3^	0.20 (0.006)	-0.07	-0.14, -0.008	-0.07	-0.15, -0.007
**Age 60-69**					
Continuous		-0.001	-0.005, 0.002	-0.001	-0.005, 0.002
Clinical cut-off					
<18.5	77 (7.03)	0.02	-0.02, 0.08	-0.006	-0.11, 0.10
18.5-25	775 (70.31)	Reference		Reference	
25-30	219 (19.05)	0.02	-0.02, 0.06	0.007	-0.03, 0.05
≥30	48 (4.46)	-0.04	-0.12, 0.04	-0.03	-0.11, 0.04
Annual change^3^		0.007	-0.10, 0.11	0.006	-0.10, 0.11
**Age 70-85**					
Continuous		0.002	-0.006, 0.01	0.002	-0.006, 0.01
Clinical cut-off					
<18.5	98 (9.61)	0.03	-0.04, 0.09	0.04	-0.05, 0.14
18.5-25	887 (76.07)	Reference		Reference	
25-30	186 (12.12)	0.04	-0.009, 0.08	0.04	-0.008, 0.08
≥30	32 (2.20)	0.04	-0.11, 0.19	0.03	-0.10, 0.15
Annual change^3^		-0.08	-0.27, 0.11	-0.08	-0.28, 0.11

1 Predicted BMI from the two-step regression approach on self-reported history of current BMI

2 Additionally adjusted for interval in years between age 25 and age at examination

3 Difference between self-reported BMI at age 25 years and BMI measured at examination divided by interval time in years

### Associations between overweight trajectories and telomere length

Finally, we estimated LTL differences across life course trajectories based on overweight status at 25 years and at examination. Compared to those who had BMI <25 kg/m2 at age 25 and at examination, shorter LTL was observed among individuals aged 30-40 years who were overweight at age 25 regardless of current overweight status (Table 4). LTL was shorter among those who were overweight at age 40-50, regardless of overweight status at age 25. Interestingly, a 12% increase in LTL (1 to 24%) was observed among those who were overweight at age 25 and had normal weight at age 50-60 years. No association was found among those aged 60 and over. A significant interaction was found between overweight trajectory and current age (P_interaction_=0.0007).

**Table 4:** Associations between overweight trajectories and telomere length, stratified by age at examination

**Age at examination**	**N (%)**	**Mean BMI (SE)**		**Mean LTL(SE)**	**Log LTL difference^1^**	
		**At age 25**	**At examination**		**β**	**95% CI**
**Age 30-39**						
Non-overweight/non-overweight	393 (36.07)	20.81 (0.11)	22.14 (0.10)	1.13 (0.02)	Ref	
Non-overweight /overweight	368 (29.97)	22.44 (0.12)	28.97 (0.21)	1.13 (0.02)	-0.009	-0.04, 0.02
Overweight/non-overweight	26 (2.16)	27.09 (0.31)	23.52 (0.25)	0.99 (0.03)	-0.12	-0.20, -0.05
Overweight/overweight	395 (31.80)	29.53 (0.23)	33.69 (0.39)	0.99 (0.04)	-0.13	-0.19, -0.02
**Age 40-49**						
Non-overweight/non-overweight	320 (28.46)	20.59 (0.12)	22.10 (0.12)	1.12 (0.02)	Ref	
Non-overweight /overweight	571 (41.46)	21.88 (0.10)	29.45 (0.24)	1.05 (0.02)	-0.06	-0.11, -0.02
Overweight/non-overweight	37 (3.58)	27.09 (0.31)	23.29 (0.31)	1.13 (0.05)	0.006	-0.08, 0.09
Overweight/overweight	360 (26.50)	28.83 (0.36)	34.30 (0.58)	1.05 (0.03)	-0.07	-0.13, -0.009
**Age 50-59**						
Non-overweight/non-overweight	239 (26.75)	20.28 (0.13)	22.46 (0.13)	0.99 (0.02)	Ref	
Non-overweight /overweight	492 (49.13)	21.97 (0.13)	29.45 (0.24)	0.98 (0.02)	-0.005	-0.05, 0.04
Overweight/non-overweight	19 (1.49)	27.12 (0.61)	23.44 (0.42)	1.14 (0.07)	0.12	0.01, 0.24
Overweight/overweight	225 (22.63)	28.52 (0.32)	33.79 (0.69)	1.01 (0.02)	0.03	-0.03, 0.08
**Age 60-69**						
Non-overweight/non-overweight	253 (23.86)	20.52 (0.14)	22.54 (0.14)	0.96 (0.02)	Ref	
Non-overweight /overweight	649 (54.02)	21.44 (0.09)	30.33 (0.25)	0.96 (0.02)	-0.01	-0.06, 0.03
Overweight/non-overweight	16 (1.01)	26.25 (0.37)	23.35 (0.50)	0.89 (0.07)	-0.05	-0.22, 0.10
Overweight/overweight	251 (21.11)	27.66 (0.23)	33.16 (0.38)	0.96 (0.02)	0.001	-0.05, 0.06
**Age 70-85**						
Non-overweight/non-overweight	393 (32.49)	20.64 (0.12)	22.23 (0.15)	0.87 (0.02)	Ref	
Non-overweight /overweight	676 (53.88)	21.24 (0.08)	29.64 (0.18)	0.86 (0.01)	-0.009	-0.04, 0.03
Overweight/non-overweight	39 (2.15)	28.05 (0.60)	22.93 (0.41)	0.87 (0.05)	0.02	-0.10, 0.13
Overweight/overweight	179 (11.48)	27.57 (0.20)	30.80 (0.37)	0.88 (0.03)	0.03	-0.04, 0.10

Note: Trajectories reflect overweight status at age 25, followed by that at current age, e.g. non-overweight/overweight refers to non-overweight at age 25 and overweight at current age.

1 All models were adjusted for sex, race/ethnicity, education, smoking status, alcohol intake, and vigorous physical activity.

## DISCUSSION

We found decreased LTL with increasing BMI, waist circumference and percent body fat, and among those with excess adiposity compared to those without. Higher BMI and being obese at age 25 was associated with lower LTL at age 30-40. Among those who were overweight at age 25, LTL decreased among those who became non-overweight at age 30-40, and increased among those who were non-overweight at age 50-60, compared to those who were non-overweight both at age 25 and at current age.

Excess adiposity may lead to telomere shortening through inflammation and generation of oxidative stress [19]. As an endocrine organ [20], adipose tissue may promote inflammation through directly secreting pro-inflammatory mediators and through increased levels of adipokines, particularly leptin [21]. Pro-inflammatory properties of leptin have been shown [21], with binding to its receptor leading to activation of signalling pathways involved in chronic inflammation and age-related diseases such as JAK/STAT and NF-κB [22]. Corroborating a role of obesity in ageing, we found further evidence linking excess adiposity and shorter telomere length which echoed some previous findings [15, 23–26]. Despite the suggested association, a Mendelian randomisation analysis comprising Danish populations found no evidence of a causal association between BMI and telomere length [24]. Insufficient sample size and mediation of the effect by inflammation as shown by genetically-determined C-reactive protein were argued to have explained this null association [24]. Additionally, many studies assessed adiposity and LTL in a linear fashion [14, 27]. In a systematic analysis of environmental exposures and LTL in the NHANES, linear associations between BMI, waist circumference and body fat were also noted although only trunk fat remained significant after adjustment for multiple comparison [27]. Additionally, associations with adiposity history were not examined in the above study. Given nonlinear associations between BMI and health outcomes such as mortality [28], the use of clinically meaningful categories of adiposity may be more useful for translation to clinical and public health measures.

In addition to their cross-sectional association, the longitudinal correlation between adiposity and telomere length is a subject of controversy. A standard deviation increase in waist circumference over 6 years was linked to a 40 base pair decrease in LTL during the same period in a Dutch study [29]. Nevertheless, a Danish study including over 4,500 Caucasians and a cohort comprising older German populations showed a lack of association between weight change and LTL attrition between two measurements 10-year and 8-year apart, respectively [30, 31]. There is also a lack of understanding regarding any critical or sensitive periods in which adiposity affects telomere length in later life. In the Health and Retirement Study, a positive association was shown between being overweight or obese in the past 16 years and current telomere length among middle-aged and older adults [32]. In our study, however, we found associations between BMI at early adulthood and current telomere length were limited to those younger than 60. These discrepancies further indicate the importance of understanding the dynamics between adiposity and biological ageing throughout lifespan and whether such association affects age-related diseases.

Differing impacts of weight loss were indicated by longer LTL among currently non-overweight individuals aged 50-60 who used to be overweight at age 25 compared to those who stayed non-overweight at both time points, and an opposing association for individuals currently aged 30-40. Corroborating the former, restored telomere length among overweight and obese individuals has been suggested following specific weight loss interventions, including bariatric surgery [33] and bioenteric intragastric balloon (BIB) in adults [34], and lifestyle modification in adolescents [35]. Nevertheless, a positive association between weight loss and LTL was observed among BIB but not bariatric surgery patients [33, 34, 36] and no alteration of telomere length was observed following lifestyle modification in adults [37, 38]. Except one study which followed the participants until 10 years after undergoing bariatric surgery [33], these previous findings had relatively short-term follow-up (6 months to 4.5 years), which may have limited the sensitivity in observing changes secondary to weight loss. Additionally, our varying results in different age groups may support varying roles of stages at which weight loss takes place as indicated by discrepancies of prior findings among adolescents and adults [35]. Alternatively, the weaker associations we observed between overweight trajectories in older age groups may imply that any effect of adiposity on telomere length is less apparent with longer intervals. Considering these potential explanations, further clarification regarding the impact of life course adiposity on ageing is necessary.

The strength of this study lies on the nationally representative sample included in the NHANES surveys. We were able to assess different measures of adiposity and take into account potential confounders and co-morbid metabolic disorders. Spurious correlations may be of concern when performing multiple comparisons as shown in our study. However, we planned our analyses based on prior evidence and our results are explicable by suggested biological pathways and findings from other studies [39]. Therefore the observed association is unlikely to be spurious. One of the limitations of this study is that information was based on cross-sectional assessments. Whilst we were able to evaluate past BMI history and assess trajectories of overweight, our findings only implied association rather than causation. Recall bias may lead to misclassification when using self-reported data. However, we predicted BMI at age 25 by taking into account discrepancy between current measured and self-reported BMI, and found similar results. Another limitation is that telomere length may be altered in carcinogenesis [40]. Although our findings were robust when adjusted for history of cancer, residual confounding may have occurred due to lack of information on cancer incidence.

In summary, our cross-sectional analysis revealed that excess adiposity corresponds to shorter telomeres. Weight loss among overweight individuals during early adulthood may be associated with short-term telomere shortening, and longer-term telomere elongation. Our findings provide the first indication that the relationship between obesity and telomere length changes over an individual's life course. Studies using objective measures of obesity should be conducted to confirm our conclusions.

## MATERIALS AND METHODS

### Study population

The National Health and Nutrition Examination Survey (NHANES) is a cross-sectional health survey conducted by the National Center for Health Statistics (NCHS) in representative samples of the non-institutionalized U.S. population [41]. The NCHS Institutional Review Board (IRB) approved the study and informed consent was obtained from participants. Participants were selected through a multistage stratified, clustered probability sampling. The survey included an interview conducted at home and an extensive physical examination, which included a blood sample taken in a mobile examination center (MEC). This study was based on the continuous NHANES 1999-2002, which included 21,004 participants of all ages. From this population, we selected men and women aged 20 years and older who had measurements of BMI and waist circumference (N=8,889). Pregnant women, determined by a positive urine pregnancy test and/or self-reported at the MEC, were excluded (N=547). Among the remaining participants, a total of 7,027 individuals had measurements of leukocyte telomere length. We further excluded those with missing values for other covariates: education level, smoking status, alcohol consumption, and physical activity (N=19). Age and race/ethnicity distribution did not differ between those with missing values and the final study population with complete data (N=7,008).

### Telomere length assay

DNA was extracted from whole blood and stored at−80°C. The telomere length assay was performed in the laboratory of Dr. Elizabeth Blackburn at the University of California, San Francisco, using the quantitative polymerase chain reaction (PCR) method to measure telomere length relative to standard reference DNA (T/S ratio), as described in detail elsewhere [42, 43]. Each sample was assayed 3 times on 3 different days. The samples were assayed on duplicate wells, resulting in 6 data points. Sample plates were assayed in groups of 3 plates, and no 2 plates were grouped together more than once. Assay runs with 8 or more invalid control wells were excluded from further analysis (<1% of runs). Control DNA values were used to normalize between-run variability. Runs with more than 4 control DNA values falling outside 2.5 standard deviations from the mean for all assay runs were excluded from further analysis (<6% of runs). For each sample, any potential outliers were identified and excluded from the calculations (<2% of samples). The mean and standard deviation of the T/S ratio were then calculated normally. The inter-assay coefficient of variation was 6.5%. A logarithmic transformation of T/S ratio was performed due to its skewed distribution.

### Adiposity assessment

All body measurements were performed using standardized methods and equipment [41]. Current body mass index (BMI) was calculated from measured weight and height. Weight was measured with an electronic weight scale in pounds and automatically converted to kilograms. Participants only wore underwear, disposable paper gowns and foam rubber slippers. Standing height was measured with a fixed stadiometer to the nearest 1 mm. Waist circumference was measured at the high point of the iliac crest at minimal respiration using a steel measuring tape to the nearest 1 mm [41]. Fat mass was determined through a whole body DXA scans with a Hologic QDR-4500A fan-beam densitometer (Hologic, Inc., Bedford, Massachusetts). Hologic software version 8.26:a3* was used. The densitometer scanned participants with an x-ray source using fan-beam scan geometry while the participants were positioned supine on the tabletop. Percent body fat was calculated by dividing total fat mass by total DXA mass (fat mass and fat-free mass) and multiplying by 100.

BMI was also divided into four categories (<18.5, 18.5-25, 25-30, and ≥30 kg/m2) according to the National Health Institute (NIH) guidelines and dichotomously into non-overweight and overweight using 25 kg/m2 as a cut-off point [44]. Excess abdominal obesity was defined as waist circumference >102 cm in men and >88 cm in women based on guidelines published by international organizations including the experts in the Adult Treatment Panel (ATP) under the National Cholesterol Education Program (NCEP) [45]. According to the World Health Organization (WHO) guidelines, [46] percent body fat ≥25 in men and ≥35 women were defined as having excess body fat. Self-reported weight and height at 25 years was collected for those aged 30 years and older and BMI at age 25 was calculated. The difference between in BMI between 25 and the age at survey measurement was calculated and divided by the number of years between the two time points to produce the annual change in BMI.

### Assessment of potential confounders

Several demographics and lifestyle factors were considered as potential confounders. Age was assesses as a continuous variable in the initial analysis and subsequently grouped into 20-29, 30-39, 40-49, 50-59, 60-69, and 70-85 years. Race/ethnicity was categorized into Non-Hispanic white, Non-Hispanic black, Mexican-American, and other. We classified educational attainment as less than high school, high school equivalent, and higher than high school. This information provided a proxy of socio-economic status which has been reported to impact ageing-related outcomes [47, 48]. We defined current smokers as those who had reported a history of smoking at least 100 cigarettes during their lifetime and, at the time of the interview, reported smoking either every day or some days. Former smokers were those who reported smoking at least 100 cigarettes during their lifetime but currently did not smoke. Never smokers were those who had not smoked 100 cigarettes during their lifetime. Vigorous physical activity (yes, no) was described as reporting 3 or more physical activities of at least 6 metabolic equivalents (METs) per week, each with duration of at least 10 minutes [49]. Frequency of alcohol consumption was collected during interview and categorized into never (had less than 12 alcohol drinks in the entire life), up to once a week, 2-3 times a week, and 4 times a week or more. A self-reported history of cancer diagnosis (yes, no) was based on a positive response to the question “Have you ever been told by a doctor or other health care professional that you had cancer or a malignancy of any kind”.

In addition to demographics and lifestyle, other metabolic disorders were taken into account given their associations to obesity and age-related disease such as cardiovascular disease. The ATP-III definition of metabolic syndrome apart from obesity was used to define presence of other metabolic disorders (yes, no) [45]. Hypertension was defined by blood pressure of ≥130/≥85 mmHg or any use of antihypertensive drugs. Diabetes was defined as fasting glucose ≥110 mg/dL (5.55 mmol/L) or any use of insulin or glucose-lowering drugs. Any HDL-cholesterol levels <40 mg/dL (1.04 mmol/L) for men or <50 mg/dL for women (1.29 mmol/L) were considered as low. Triglyceridemia was defined as any levels of fasting triglycerides ≥150 mg/dL (1.69 mmol/L). Blood pressure was measured with mercury manometer and the average of the second and third blood pressure measurements was taken. Plasma glucose levels were measured by using a hexokinase method. Blood lipids were measured enzymatically [41].

### Statistical analysis

Survey-weighted linear regression was performed to assess differences in log-transformed T/S ratio by different levels of current adiposity measures, using the weights specific for the genetic subsample [41, 42]. The percentage change in LTL was estimated by multiplying the parameter estimate by 100 [42]. First, we used models adjusted for age as a continuous variable, sex and race/ethnicity to assess any difference in log-transformed LTL by current measured BMI, waist circumference, and percent body fat as continuous variables. A similar analysis was performed using the four categories of BMI and dichotomous categories of waist circumference and percent body fat. Additionally, we also assessed overweight status by BMI as a dichotomous variable (</≥25 kg/m2). The final models were further adjusted for variables which have been linked with both adiposity and ageing-related outcomes including education, smoking status, alcohol intake, and vigorous physical activity. To additionally check if this association was affected by metabolic disorders and cancer, we adjusted the model for presence of other metabolic disorders and cancer diagnosis.

To further characterise the cross-sectional associations between adiposity and telomere length, we used fully adjusted quantile regression models assessing difference in quantiles of LTL with increasing BMI, waist circumference, and percent body fat. This analysis predicted changes in a given quantile, e.g. the 90th quantile of outcome variables instead of their mean, allowing a more comprehensive view of the relationship between predictors and outcomes [50, 51]. Wald test was used to test equality of coefficients across quantiles [52].

We estimated the associations between self-reported BMI at age 25 and LTL at examination for those aged 30 and older. Participant age at examination varied in the NHANES. Therefore, we adjusted our models for current age in the analysis and we further stratified our analysis by age group. Finally, to further examine the impact of adiposity changes over the life course on biological ageing, we created four indicators of adiposity trajectories based on the combination between overweight status at age 25 and at examination based on the dichotomous BMI cut-off (non-overweight: <25 kg/m2 and overweight: ≥25 kg/m2). Difference in LTL across different trajectories (non-overweight/non-overweight, non-overweight/overweight, overweight /non-overweight, and overweight/overweight) was estimated in regression models treating those who had normal weight both at age 25 and at examination (non-overweight/non-overweight) as the referent category. A test for interaction was performed by incorporating the product term between BMI at age 25 or overweight trajectories and age categories.

Because BMI at age 25 was only available from self-reported data, we performed a sensitivity analysis addressing the discrepancy between self-reported and measured BMI. First, we took the estimate or regression coefficient from a linear model regressing measured BMI over self-reported BMI at current age, which was regarded to reflect the difference between objectively measured and self-reported BMI. This estimate was multiplied by self-reported BMI at age 25 as an estimation of objectively measured BMI at age 25. We subsequently assess LTL based on the predicted BMI at age 25.

The NHANES data was prepared with Statistical Analysis Software (SAS) release 9.3 (SAS Institute, Cary, NC) and survey-weighted analyses were performed with the survey package of R version 3.1.2 (R Foundation for Statistical Computing, Vienna, Austria). The R quantreg package incorporating sampling weight replicates were used to obtain quantile regression estimates, with ninety-five percent confidence intervals (95% CI) obtained using 100 bootstrap re-samples.
